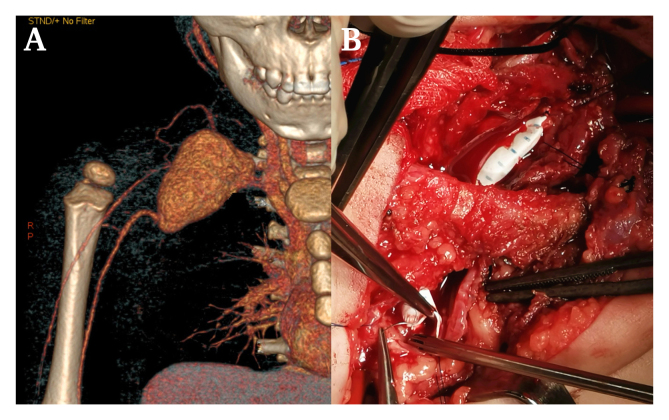# Huge Paediatric Subclavian Artery Aneurysm

**DOI:** 10.1016/j.ejvsvf.2022.04.006

**Published:** 2022-04-29

**Authors:** Yu Yang, Gang Shen

**Affiliations:** Department of Vascular Surgery, Tsinghua University Affiliated Beijing Tsinghua Changgung Hospital, Beijing, China; Department of Interventional Radiology and Vascular Anomalies, Capital Institute of Paediatrics Affiliated Children's Hospital, Beijing, China

A 19 month old boy presented with a one month history of painless pulsatile mass in the right supraclavicular fossa without traumatic history. He had no significant congenital diseases and no other aneurysms. Contrast enhanced CT showed a 40 x 32 x 42 mm saccular aneurysm of the right subclavian artery (A). Covered stenting was attempted but the distal right axillary artery could not be crossed with the wire using a transfemoral approach. Open surgery was performed under general anaesthesia (B). The aneurysm was located behind the right clavicle, and the proximal part of the right subclavian artery and the distal right axillary artery were controlled. A polytetrafluoroethylene terephthalate graft (5 mm diameter) was used without clavicular resection. The patient was sent to the paediatric ICU and discharged with no complications. His right radial pulse was palpable, and graft patency was confirmed by duplex ultrasound.

## Acknowledgements

We would like to thank Dr LiLong, MD, from the Department of General Surgery and Dr ZhangHui, MD, from the Department of Cardiac Surgery at CIP for their fantastic surgery.

## Publication consent

Written publication consent for case details and images was obtained from the guardian prior to preparation and submission of this manuscript and is available upon request.

## Conflict of interest

None.

## Funding

None.

**Figure:**